# Markerless dynamic tumor tracking (MDTT) radiotherapy using diaphragm as a surrogate for liver targets

**DOI:** 10.1002/acm2.14161

**Published:** 2023-10-03

**Authors:** Maryam Rostamzadeh, Steven Thomas, Marie‐Laure Camborde, Tania Karan, Mitchell Liu, Roy Ma, Ante Mestrovic, Bradford Gill, Isaac Tai, Alanah Bergman

**Affiliations:** ^1^ Department of Physics and Astronomy University of British Columbia Vancouver British Columbia Canada; ^2^ Medical Physics Department BC Cancer‐Vancouver Vancouver British Columbia Canada; ^3^ Radiation Oncology Department BC Cancer‐Vancouver Vancouver British Columbia Canada; ^4^ Radiation Therapy Department BC Cancer‐Vancouver Vancouver British Columbia Canada

**Keywords:** correlation model, diaphragm, dynamic tumor tracking, liver, markerless, SABR, tracking structure, vero4DRT

## Abstract

**Purpose:**

To assess the feasibility of using the diaphragm as a surrogate for liver targets during MDTT.

**Methods:**

Diaphragm as surrogate for markers: a dome‐shaped phantom with implanted markers was fabricated and underwent dual‐orthogonal fluoroscopy sequences on the Vero4DRT linac. Ten patients participated in an IRB‐approved, feasibility study to assess the MDTT workflow. All images were analyzed using an in‐house program to back‐project the diaphragm/markers position to the isocenter plane. ExacTrac imager log files were analyzed. Diaphragm as tracking structure for MDTT: The phantom “diaphragm” was contoured as a markerless tracking structure (MTS) and exported to Vero4DRT/ExacTrac. A single field plan was delivered to the phantom film plane under static and MDTT conditions. In the patient study, the diaphragm tracking structure was contoured on CT breath‐hold‐exhale datasets. The MDTT workflow was applied until just prior to MV beam‐on.

**Results:**

Diaphragm as surrogate for markers: phantom data confirmed the in‐house 3D back‐projection program was functioning as intended. In patients, the diaphragm/marker relative positions had a mean ± RMS difference of 0.70 ± 0.89, 1.08 ± 1.26, and 0.96 ± 1.06 mm in ML, SI, and AP directions. Diaphragm as tracking structure for MDTT: Building a respiratory‐correlation model using the diaphragm as surrogate for the implanted markers was successful in phantom/patients. During the tracking verification imaging step, the phantom mean ± SD difference between the image‐detected and predicted “diaphragm” position was 0.52 ± 0.18 mm. The 2D film gamma (2%/2 mm) comparison (static to MDTT deliveries) was 98.2%. In patients, the mean difference between the image‐detected and predicted diaphragm position was 2.02 ± 0.92 mm. The planning target margin contribution from MDTT diaphragm tracking is 2.2, 5.0, and 4.7 mm in the ML, SI, and AP directions.

**Conclusion:**

In phantom/patients, the diaphragm motion correlated well with markers’ motion and could be used as a surrogate. MDTT workflows using the diaphragm as the MTS is feasible using the Vero4DRT linac and could replace the need for implanted markers for liver radiotherapy.

## 1 INTRODUCTION

The goal of radiotherapy is to deliver radiation to a target volume while sparing normal tissues. This can be a challenge due to several sources of uncertainty in the radiotherapy treatment process. One intractable source of uncertainty is intra‐fractional respiratory motion for tumors located near the diaphragm.^1^ If not accounted for in the target margins (i.e., using motion encompassing or ``ITV’’ methods), this may lead to under dosing of the radiotherapy target. However, increasing margins to account for target motion increases the amount of overlap with nearby healthy organs at risk, which is also not ideal.^2^ Liver tumor motion is greatly impacted by respiration, particularly in the superior–inferior (SI) direction.^2^, ^3^ Rodgerson et al.^4^ assessed the variability of liver tumor motion (represented by fiducial marker surrogates) during liver radiotherapy in 26 patients over 85 fractions. The reported mean (range) of liver motion in the SI direction was 18 mm (9–32 mm). Liang et al.^5^ analyzed the tumor motion in 14 liver cancer patients over 66 fractions. They reported the median (range) of liver motion amplitudes of 11.9 mm (5.1−17.3 mm) in the SI direction.

Different motion management techniques have been developed for treating tumors affected by respiration; motion‐encompassing methods, breath holding techniques, respiratory gating, and dynamic tumor tracking (DTT).^2^, ^6^, ^7^, ^8^ Real‐time DTT is the most recent approach where the radiotherapy beam follows the tumor motion throughout the entire respiratory cycle. The advantage of DTT over simpler motion encompassing techniques is that smaller target margins are required as the motion is being compensated for daily at the treatment unit. In addition, daily variations in breathing motion can be compensated for in real‐time. Several commercial and research systems are available that can perform DTT.^9^, ^10^, ^11^, ^12^, ^13^, ^14^, ^15^, ^16^, ^17^, ^18^, ^19^

Common to all the commercial systems, kV x‐ray fluoroscopy is used to quantify internal target motion. However, liver tumors typically are not easily visualized with most kV x‐ray based imaging systems (e.g., planar 2D images or 3D CT) and tumor tracking methods rely on the presence of implanted radio‐opaque fiducial markers to accurately target and track the tumor.^20^, ^21^, ^36^ These markers (typically three to four short gold markers [<5 mm] or one long wire/coil >10 mm, <1 mm in diameter) are inserted prior to acquiring the pre‐planning CT simulation images. Fiducial markers are generally implanted by percutaneous needle insertion by an interventional radiologist. Disadvantages of implanting markers into patients include: potential toxicity related to the marker insertion procedure, the associated extra interventional x‐ray imaging dose, additional resources associated with the interventional procedure, and the extra time/visits required by the patient.^22^, ^23^ After implantation, technical complications may include marker migration^23^, ^24^ and possible ``seeding’’ of the needle track with malignant cells (as reported for some hepatocellular cancer patients).^25^ Some patients are ineligible for marker placement due to the location of the tumor (poor or risky needle access), or due to their general condition.

To address the disadvantages and risks associated with metal fiducial marker placement, direct‐ or markerless‐tracking is currently available for eligible lung tumors on three clinical systems; Accuray CyberKnife (Accuray Inc., Sunnyvale, CA, USA),^26^ Accuray Radixact,^27^ and Brainlab Vero4DRT (Brainlab AG, Munich, Germany and Mitsubishi Heavy Industries, Tokyo, Japan).^28^ In a clinical research trial context, the Varian TrueBeam has been used to deliver markerless lung MLC tumor tracking.^29^ A recent AAPM “Grand Challenge” (MArkerless lung target Tracking CHallenge [MATCH]) compared the performance of various clinical and research systems offering markerless tracking radiotherapy.^30^ For the experimental study, 11 systems participated, including commercial and pre‐clinical research systems. In the commercial systems, the best result for the multiple Radixact and CyberKnife submissions, and the result of the single Vero4DRT submission indicated sub‐millimeter target tracking accuracy and precision.

Recently, there has been interest in finding alternative anatomical tumor surrogates (e.g., diaphragm), that can be used in lieu of the invasive marker placement for certain clinical situations (e.g., lung and liver).^3^, ^31^, ^32^, ^33^, ^34^, ^35^ The ideal anatomical surrogate will have a linear and stable correlation with the actual treatment target. There are limited publications describing the use of soft‐tissue anatomy tracking surrogates for real‐time MDTT applied to liver radiotherapy. It should be noted that there are many instances in the literature where markerless “tumor monitoring” (passive monitoring vs. active real‐time target tracking) has been labeled as “tumor tracking.” In the context of this study, “tumor tracking” is defined as building a respiratory correlation model which allows the radiotherapy beam to actively follow the tumor target during its motion trajectory, with a 100% beam‐on duty cycle.

A MDTT module is available for direct‐lung‐target applications on the Vero4DRT linear accelerator (linac).^28^ This study investigates the efficacy of using this module in a novel manner such that instead of direct lung GTV tracking, the diaphragm is tracked as a soft tissue surrogate for superior liver (or inferior lung) targets.

This manuscript is divided into two parts. Part 1 verifies on kV images that diaphragm motion can be used as a surrogate for fiducial marker motion for locations within 8.2 cm from the liver/lung interface. Part 2 of this manuscript demonstrates that the contrast from the diaphragm is enough to build a respiratory correlation model for markerless dynamic tumor tracking radiotherapy, potentially eliminating the need for implanted fiducial markers.

## 2 METHODS

### 2.1 Vero4DRT radiotherapy linear accelerator

The Vero4DRT radiotherapy linear accelerator (Brainlab AG, Munich, Germany) is comprised of a compact, 6MV, C‐band waveguide (∼6 GHz/38 cm) situated in‐line with the target and collimation system (maximum dose rate = 500 MU/min @ 100 cm SAD). The treatment head is mounted in an O‐ring type gantry.^11^ The gantry can rotate around the patient about two axes (vertical and longitudinal) to create non‐coplanar radiotherapy treatment beams. The entire treatment head is mounted on a 2D moveable gimbal capable of pan and tilt directions which enables real time respiratory motion‐correlated DTT.^13^, ^37^, ^38^, ^39^, ^40^, ^41^ The greatest achievable mechanical deflection of the beam center is ±2.5° which corresponds to ±4.4 cm at the isocenter level in a pan and tilt direction. The Vero4DRT linear accelerator is equipped with an integrated, dual‐orthogonal kV x‐ray imaging system. The two on‐board orthogonal x‐ray tubes (field size 216 mm x 162 mm at isocenter plane) are installed into the O‐ring gantry at ±45^◦^ from the MV beam axis. These imagers enable cone‐beam CT and real‐time fluoroscopy imaging at a maximum frame rate of 15 frames/s.^40^ A ceiling mounted infrared (IR) camera system monitors a chest pad with IR reflectors which becomes an external motion surrogate.^38^ A fluoroscopy sequence is acquired while simultaneously recording the external IR motion signal which allows for the creation of a respiratory correlation model in the ExacTrac (Brainlab AG) software.^38^ ^,40,^ ^43^ The gimbal tracking is then determined by the external (chest) IR motion signal by applying the correlation model. Verification images confirm that the model of the internal motion is still valid. The Vero4DRT has a mechanical geometric accuracy (<1 mm) and tracking latency of <50 ms with IR camera tracking.^44^

### 2.2 Phantom design

**FIGURE 1:**
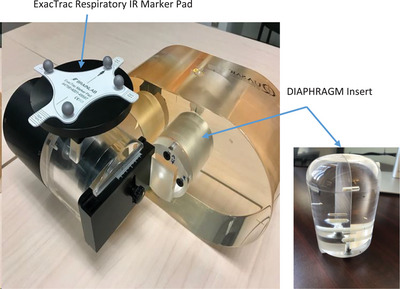
QUASAR motion phantom with IR respiratory pad and “liver” insert. Inset: photo of “LIVER/DIAPHRAGM” insert with film plane bisecting the phantom.

An in‐house, acrylic, “liver/diaphragm” phantom insert was designed and built such that it was compatible with the QUASAR (ModusQA, London, Ontario, Canada) respiratory motion phantom (Figure 1). Three gold seeds (1 mm × 3 mm) were inserted into the acrylic liver insert as fiducial markers. The respiratory motion platform offers synchronized superior–inferior (SI) motion of the insert with anterior‐posterior (AP) motion of an external “chest” platform onto which the ExacTrac respiratory IR maker pad was placed. A 3D helical CT was acquired of the phantom, and the curved dome of the phantom insert was contoured as “diaphragm.” The three gold seeds were contoured as “markers.”

### 2.3 ExacTrac kV image analysis

All fluoroscopy x‐ray images acquired in this study were extracted from the ExacTrac imaging server. There are two time points along the workflow that will acquire images; (1) during the correlation model building fluoroscopy imaging, and (2) during the verification imaging while the linac is in a gimbal tracking state. An in‐house MATLAB program (The MathWorks, Natick, MA, USA) was developed to detect the diaphragm the fiducial marker positions on raw kV images. Each 1024 × 768‐pixel image matrix was converted to a 256 gray scale intensity image. Images were pre‐processed using a median filter to remove dead pixel values. The 2D position of the diaphragm (represented as points on a line) and the implanted markers (as single points) were automatically detected in all dual‐orthogonal fluoroscopy images based on a template matching method employing normalized cross correlation. Using rotation and translation matrices (accounting for various non‐coplanar gantry and ring angles), the 3D position at isocenter of the center‐of‐mass (COM) of the diaphragm‐line and the COM of the fiducial markers was reconstructed by back projecting the orthogonal 2D positions towards the source. The intersection of the back‐projected ray traces allows for the 3D position of the objects to be located for each fluoroscopy image‐pair. From this information, a motion trace for the COM of the diaphragm and markers was calculated. The absolute distance between the COM of the diaphragm versus markers position during the respiratory cycle was reported.

### 2.4 ExacTrac MDTT log file analysis

Several text‐based log files related to the ExacTrac imaging and dynamic tumor tracking were generated during each MDTT session. These log files include: (1) the 3D position of external IR markers, (2) the correlation model parameters used to build the respiratory motion model, (3) the image‐detected and model‐predicted 3D COM position of the MTS for every correlation model‐building step and treatment verification imaging step, and (4) linac parameters such as gantry angle, ring angle, position of the moving isocenter, and the BEAM ON/OFF status for each datapoint in time. Using this information, the quality of the correlation model, and the detected versus predicted MTS tracking accuracy was assessed.

### 2.5 Part 1: Diaphragm motion as surrogate for marker motion

#### 2.5.1 Phantom study

The motion platform was programmed with a sinusoidal breathing trace (6 BPM, 22.5 mm amplitude). Dual‐orthogonal kV x‐ray fluoroscopy sequences were acquired in clinical mode using a variable frame rate customized to the detected breathing trace. Raw images were stored for off‐line analysis. The kV x‐ray detected phantom “diaphragm” structure and “markers” structure were located on the dual‐orthogonal images and a 3D motion trace at the isocenter plane was reconstructed using back projection methods (see Section 2.3).

#### 2.5.2 Patient study

An institutional review board (IRB) approved imaging‐only study was created to allow for the acquisition of x‐ray fluoroscopy images on 10 patients undergoing liver radiotherapy between June 2020 and November 2021. Patients undergoing standard‐of‐care liver stereotactic ablative radiotherapy (SABR) using a marker‐based dynamic tumor tracking (DTT) technique were offered the study. All patients had previously implanted gold seed markers in the liver (1 mm × 3 mm, three to four markers per patient). To be eligible, the markers implanted in the liver must be located within 10 cm of the diaphragm. Patient characteristics are found in Table 1. The kV x‐ray detected phantom “diaphragm” structure and fiducials structure were located on the dual‐orthogonal fluoroscopy images and a 3D motion trace of the COM of each structure at the isocenter plane was reconstructed using back projection methods (see Section 2.3).

**TABLE 1:** Patient and plan characteristics for MDTT imaging study.

Patient ID	Sex	Age	GTV volume (cm^3^)	PTV volume (cm^3^)	Distance between COM of markers to diaphragm dome in SI direction (mm)
1	F	81	4.7	41.2	37
2	M	78	2.3	28.9	27
3	F	85	23.1	95.6	41
4	M	67	112.5	304.8	47
5	M	72	237.6	388.7	82
6	M	77	2.8	33.5	34
7	M	63	191.3	384.7	43
8	M	73	9.1	57.6	22
9	M	73	35.8	125.15	34
10	M	70	7.3	53.4	17
Mean (range)	–	74 (63–85)	62.7 (2.3–237.6)	151.4 (28.9–388.7)	38.4 (17–82)

### 2.6 Part 2: Diaphragm as tracking structure for markerless dynamic tumor tracking (MDTT) radiotherapy

A markerless dynamic tumor tracking (MDTT) module is available on the Vero4DRT and was originally designed for lung gross tumor tracking. This study applied this module in a novel manner such that the diaphragm became an alternative soft tissue tracking target.

#### 2.6.1 Markerless dynamic tumor tracking (MDTT) workflow

##### Soft‐tissue surrogates: defining the markerless tracking structure (MTS) in TPS

**FIGURE 2:**
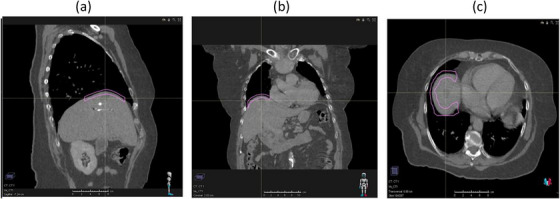
Diaphragm (pink) contoured on (a) sagittal, (b) coronal, and (c) axial CT view in TPS.

The ExacTrac imaging software requires the import of a planning CT imaging dataset and a CT structure set (with contours relevant to dynamic tumor tracking). These structures were contoured in the treatment planning system (RayStation v.7, RaySearch Medical Laboratories AB, Stockholm, Sweden). The CT dataset used at this center is a breath‐hold exhale scan, although any CT image that captures any phase of the respiratory cycle could be used. A soft tissue “Markerless Tracking Structure” (MTS) was contoured in the TPS, and it was this structure that was exported to the ExacTrac imaging system on the Vero4DRT (Figure 2).

One of the challenges encountered during this study was how to provide an optimal MTS that maximized detection by the Vero4DRT/ExacTrac module. The following features of the MTS demonstrated success at the unit.

1. Contour the diaphragm‐lung interface using lung windows setting;
2. Contour the diaphragm such that the superior surface follows the interface between the liver and lung and the contour extends 3–5 mm into the liver tissue;
3. Contour the diaphragm in both the coronal and sagittal direction to capture a “cap”‐looking structure in 3D;
4. End the contour at least 2 cm from any other soft‐tissue boundaries (e.g., chest wall or mediastinum).

##### Markerless dynamic tumor tracking (MDTT) at the Vero4DRT

**FIGURE 3:**
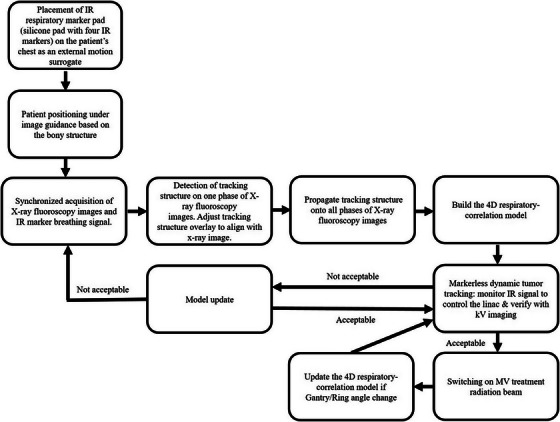
Markerless dynamic tumor tracking workflow.

The MDTT workflow at the unit (Figure 3) started with the placement of an infrared (IR) respiratory marker pad (silicone pad with four IR markers) on the patient body area as an external motion surrogate. Internal motion was monitored via a dual‐orthogonal fluoroscopy sequence. This “model‐building” sequence lasted 20–40 s (or approximately four breathing cycles). The framerate was variable and was guided by the external breathing trace to ensure sufficient sampling (∼5 frames per second).

The 3D diaphragm MTS was projected onto one set of 2D kV fluoroscopy image pairs presented by the ExacTrac software. The user adjusted the position of the projected contour overlay to match the underlying image of the diaphragm. The structure was then auto propagated onto the remaining fluoroscopy respiratory sequence. The ExacTrac system built a respiratory correlation (prediction) model connecting the 3D projected diaphragm center‐of‐mass (COM) position to the external IR respiratory signal.^28^ Once the correlation model was established, the linac motion for gimbal‐based dynamic tumor tracking was determined by monitoring the external surrogate.

**FIGURE 4:**
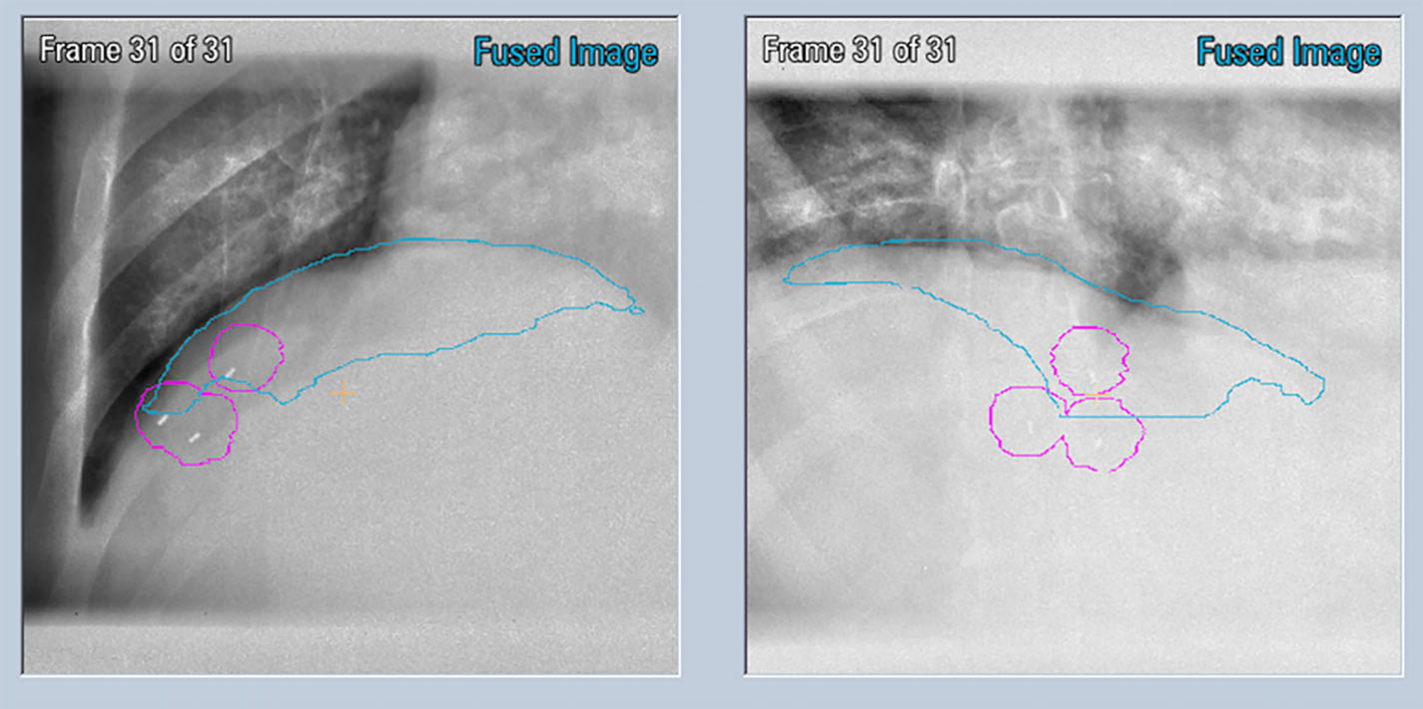
ExacTrac dual‐orthogonal images acquired on a liver study patient. Overlays include auto‐detected diaphragm contour (blue) and reference fiducial markers (with 5 mm margin) (pink).

For validation of the prediction model during the tumor tracking treatment, the diaphragm position was detected on orthogonal x‐ray imaging acquired at a user‐defined reduced framerate (∼1 Hz), see Figure 4. The COM of the detected tracking structure position on the kV x‐ray image was compared quantitatively to the predicted (correlation model) position (overlaid on kV images). During radiotherapy delivery, if the discrepancy between the predicted and detected tracking structure exceeded a user‐defined tolerance (e.g., 3 mm), the MV beam was automatically withheld, providing an opportunity for the user to either update or rebuild the correlation model.

At each new treatment beam position (delivery technique is static field step‐and‐shoot IMRT), the unit acquired an updated *“*detection template” by taking a short kV fluoroscopy series (∼10 images) so that the changing shape/size of the 2D projected tracking structure with gantry/ring angle could be updated in the model. This also allowed the user to make any fine adjustments to the MTS position prior to each beam‐on.

#### 2.6.2 Phantom study

A treatment plan consisting of a single anterior field with three MLC‐shaped segments (each centered on one of the three embedded gold fiducial markers) was created on the in‐house, acrylic, “liver/diaphragm” phantom described in Section 2.2. Each aperture had an area of approximately ∼1.5 cm^2^. GAFChromic‐EBT3 film (Ashland ISP Advanced Materials, NJ, USA) was placed in the coronal plane of the phantom to measure the dose delivered to the target area. The treatment plan was delivered under both static and MDTT delivery conditions on the Vero4DRT (using the “diaphragm” as the markerless tracking structure). All dual‐orthogonal kV fluoroscopy images and ExacTrac log files were collected for independent analysis.

##### Respiratory‐correlation model building step

The quality of the respiratory tracking *correlation model* was assessed by reporting the mean absolute deviation and standard deviation between the kV detected diaphragm COM positions and the fitted prediction model curve as reported by ExacTrac.

##### MDTT verification imaging step

The quality of the *dynamic tracking verification* is reported as the mean absolute deviation and standard deviation between the 3D position of the detected and the predicted position of the COM of the diaphragm tracking structure while the linac is in the gimbal tracking state.

Dose profiles and a 2D‐gamma comparison^45^ were used to compare the static versus MDTT film dosimetry.

#### 2.6.3 Patient study

The institutional review board (IRB) approved imaging‐only study described prior included a component to assess the feasibility of MDTT based on tracking the diaphragm MTS. The MDTT imaging workflow was performed up until the point just prior to MV beam‐on. In this state, dynamic tracking was enabled, and the gimbal position was under the control of the external motion detected by IR marker pad position. Verification images were acquired every 1 s to confirm that the 4D motion‐correlation model was valid. The ExacTrac x‐ray fluoroscopy images and log files acquired as part of the MDTT workflow were collected for analysis.

Respiratory‐correlation model building step

The quality of the respiratory correlation model was assessed by reporting the mean absolute deviation and standard deviation between the kV detected diaphragm COM positions and the fitted prediction model curve as reported by ExacTrac.

MDTT verification imaging step

The quality of the dynamic tracking verification is reported as the mean absolute deviation and standard deviation between the position of the detected and the predicted position of the COM of the diaphragm tracking structure while the linac is in the gimbal tracking state.

It is important to note that no MV beam was delivered to the patients in this imaging‐only study.

#### 2.6.4 Planning target margin estimation

According to ICRU Report 62^46^ setup and organ positional uncertainties should be included into the treatment planning process by taking a margin around the CTV, defining the PTV. In this study we have used the van Herk et al.^47^ formula, that is, calculated to achieve a minimum cumulative dose received by the CTV of at least 95% of the prescribed dose for 90% of patients. In a later overview paper, van Herk^48^ also discussed the effect of respiration and offered a practical example for calculating the uncertainty for multiple patients having multiple data‐points. This formula considered the systematic and random errors that occur during the delivery of radiation treatment. A systematic error was essentially a treatment preparation error and was introduced into the chain during the process of positioning, simulation, or target delineation. This error, if uncorrected, would affect all treatment fractions uniformly. A random error, on the other hand, was a treatment execution error, was unpredictable, and varied with each fraction. Systematic errors shifted the entire dose distribution away from the CTV, while random errors blurred this distribution around the CTV. The standard deviation (SD) of the mean data‐value per patient was an estimate for the SD of the systematic error, Σ. The root mean square (RMS) of the SDs gave the random error, *σ*, for each fraction. This information was used to estimate the PTV margin to be applied to markerless dynamic tumor tracking applications.

## 3 RESULTS

### 3.1 Part 1: Diaphragm motion as surrogate for fiducial marker motion

#### 3.1.1 kV image analysis: Correlation of diaphragm versus marker motion

**FIGURE 5:**
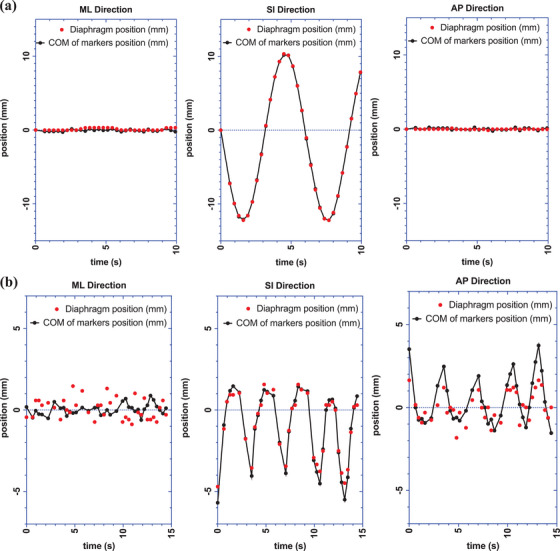
kV image back‐projection in ML, SI, and AP direction of the 3D location COM of markers (black solid line) versus the diaphragm (red dots) at isocentre plane for (a) 22.5 mm programmed phantom motion, (b) Patient #1.

**FIGURE 6:**
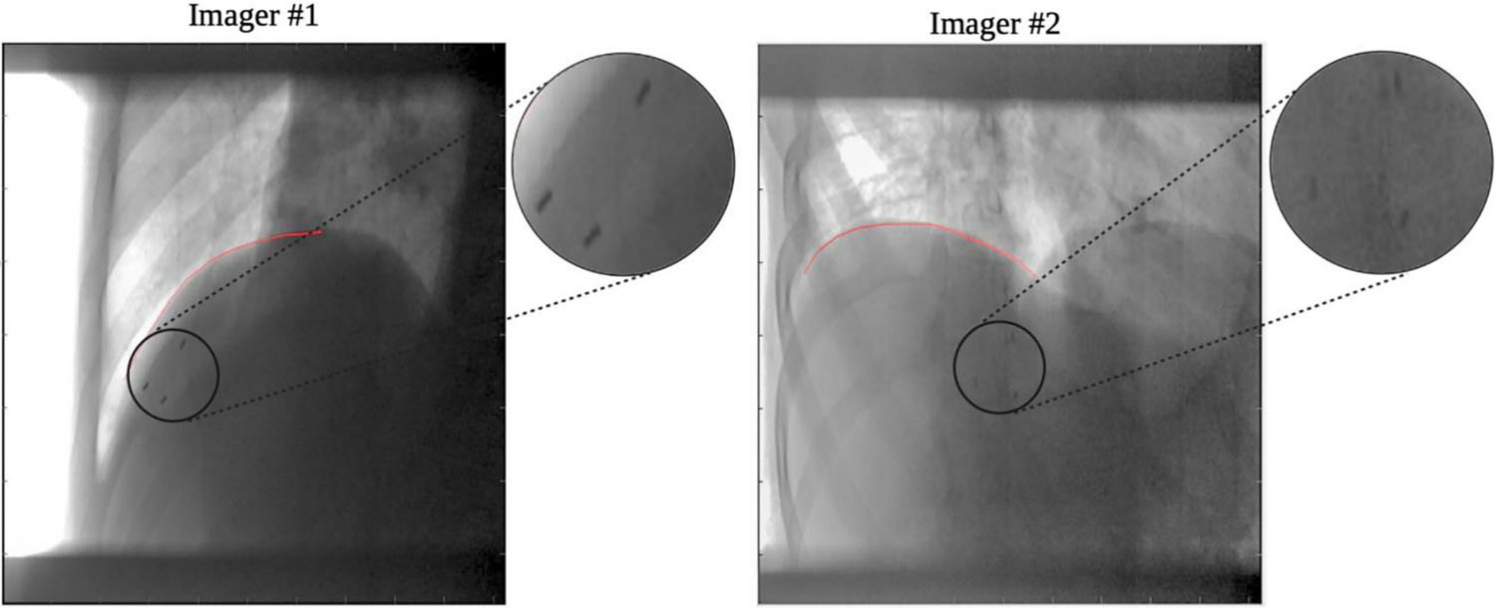
Raw orthogonal kV images showing the detected diaphragm‐lung interface (red line) and the fiducial markers (zoomed in). MATLAB code applies template‐matching and normalized cross‐correlation methods to propagate the structures on all fluoroscopy images. Coordinates from the orthogonal images are back projected to their 3D location at isocentre.

**FIGURE 7:**
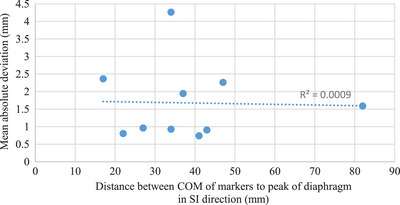
Mean absolute deviations of the COM of markers position and the diaphragm position plotted against the distance of COM of markers from diaphragm for all ten patients. The figures includes a linear regression line with an accompanying correlation coefficient (R2‐value).

From the MATLAB analysis of the dual‐orthogonal kV fluoroscopy images acquired on phantom, the motion trace of the COM of the diaphragm position versus the COM of the markers position is plotted (Figure 5a). The mean ± SD of the absolute difference between the 3D position of the COM of the “diaphragm” versus markers was 0.18 ± 0.13, 0.06 ± 0.05, and 0.12 ± 0.09 mm in the ML, SI, and AP directions, respectively. In this rigid phantom, the fiducial marker motion correlated well with the “diaphragm,” and the calculated 3D motion matched the programed motion (22.5 mm in SI direction). This confirmed that the MATLAB program image analysis and 3D back‐projection calculation was functioning correctly. In the patient study, the mean distance of the COM of the implanted liver markers relative to the diaphragm‐lung interface in the superior–inferior direction was 38 mm (range: 17–82 mm). The location of the diaphragm and the markers were identified in the raw kV images using the in‐house MATLAB analysis program (Figure 6). The 3D position of the COM of the diaphragm and the COM of the fiducial markers over the respiratory cycle for study patient #1 is shown in Figure 5b. Over the 10‐patient kV x‐ray fluoroscopy image sets acquired, the mean ± RMS of the absolute difference between the COM of markers position and diaphragm position was 0.70 ± 0.89, 1.08 ± 1.26, and 0.96 ± 1.06 mm in ML, SI, and AP directions, respectively (Table 2). No definitive correlation between the implanted marker‐diaphragm distance during respiration versus the implanted marker position was determined for this sample size of 10 patients (Figure 7).

**TABLE 2:** Mean absolute deviations between the positions of the COM of markers versus COM of diaphragm as detected on dual orthogonal kV x‐ray images in 10 liver study patients.

Patient ID	X (M/L) mean absolute deviation (mm)	X (M/L) SD (mm)	Y (S/I) mean absolute deviation (mm)	Y (S/I) SD (mm)	Z (A/P) mean absolute deviation (mm)	Z (A/P) SD (mm)
1	0.89	0.58	0.90	0.37	1.48	0.86
2	0.60	0.70	0.34	0.54	0.67	0.82
3	0.46	0.57	0.46	0.50	0.36	0.41
4	0.72	0.84	1.97	2.29	0.85	1.02
5	0.46	0.57	1.44	1.57	0.49	0.67
6	1.32	1.52	2.80	2.43	2.93	2.39
7	0.44	0.54	0.23	0.31	0.76	1.09
8	0.55	0.70	0.47	0.55	0.35	0.53
9	0.34	0.41	0.49	0.54	0.71	0.80
10	1.25	1.56	1.74	0.90	1.00	0.49
Mean	0.70	–	1.08	–	0.96	–
RMS of the SDs Random Error	–	0.89	–	1.26	–	1.06
SD of the mean Systematic Error	0.34	–	0.86	–	0.77	–

### 3.2 Part 2: Diaphragm as tracking structure for markerless dynamic tumor tracking (MDTT)

#### 3.2.1 ExacTrac MDTT log file analysis

##### Respiratory‐correlation model building step

**FIGURE 8:**
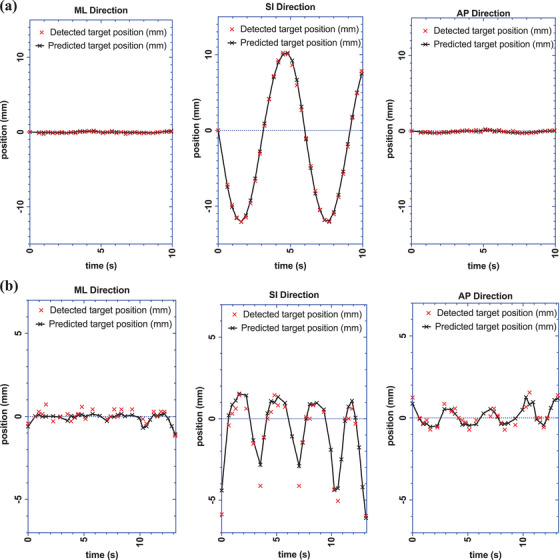
Correlation model step: ExacTrac log file detected “diaphragm” position (red ×) versus fitted correlation model (black solid line) in ML, SI, and AP directions (a) 22.5 mm programmed phantom motion, (b) Patient #1.

During the MDTT workflow applied to the phantom, the respiratory‐correlation model building step was successful. The ExacTrac‐reported mean ± SD of the absolute difference between the detected diaphragm COM position and the fitted prediction model was 0.07 ± 0.05, 0.24 ± 0.15, and 0.09 ± 0.05 mm, in the ML, SI, and AP direction, respectively. The ExacTrac‐reported detected versus prediction position of the diaphragm tracking structure is shown in Figure 8a. The ExacTrac reported peak‐to‐peak motion was 22.4 mm in SI direction. This compares well with the programmed motion (22.5 mm).

In patients, the relationship between the detected and the predicted diaphragm COM position is shown in Table 3. During the correlation model fitting step, the ExacTrac‐reported mean ± RMS of the absolute difference between the detected positions and fitted prediction‐model position at isocenter for all 10 patients was 0.50 ± 0.55, 0.71 ± 0.65, and 0.82 ± 0.93 mm in the ML, SI, and AP directions, respectively. For study patient #1, the difference between the ExacTrac‐reported detected diaphragm COM position and the fitted prediction model is shown in Figure 8b.

**TABLE 3:** Correlation model‐building step: The deviation between the detected and fitted prediction‐model positions of the diaphragm contour (MTS) at isocenter in the ML, SI, and AP direction.

Patient ID	X (M/L) mean absolute deviation (mm)	X (M/L) SD (mm)	Y (S/I) mean absolute deviation (mm)	Y (S/I) SD (mm)	Z (A/P) mean absolute deviation (mm)	Z (A/P) SD (mm)
1	0.18	0.16	0.38	0.27	0.22	0.18
2	0.46	0.66	0.69	0.96	1.00	1.32
3	0.83	0.68	0.37	0.27	1.10	0.90
4	0.43	0.32	1.34	0.73	1.72	1.42
5	0.13	0.13	1.83	1.31	0.29	0.25
6	1.55	1.26	0.51	0.46	1.55	1.65
7	0.19	0.15	0.41	0.3	0.19	0.21
8	0.28	0.32	0.42	0.24	0.37	0.26
9	0.18	0.17	0.19	0.2	0.29	0.22
10	0.74	0.45	1.00	0.69	1.49	1.05
Mean	0.50	–	0.71	–	0.82	–
RMS of the SDs						
Random Error	–	0.55	–	0.65	–	0.93

##### MDTT verification imaging step

**FIGURE 9:**
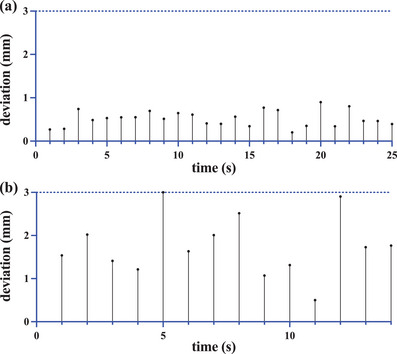
Treatment verification imaging step: absolute 3D vector difference between detected vs predicted “diaphragm” position as a function of imaging time. *Note*: Tracking error tolerance level is set to 3 mm (horizontal dashed line). (a) Phantom data, (b) Patient #1.

With dynamic tracking gimbal motion enabled during the phantom irradiation, the mean ± SD of the 3D vector difference between the ExacTrac‐reported detected “diaphragm” COM position and the predicted (and treated) COM position was 0.52 ± 0.18 mm (Figure 9a).

In the patient study, verification imaging was acquired while dynamic tracking gimbal motion was enabled during the pre‐MV beam state, however, no MV beam was actually delivered. The ExacTrac‐reported 3D absolute deviations between the kV‐detected and predicted COM of the diaphragm for patient #1 is shown in Figure 9b. The mean ± RMS of the 3D absolute difference between these data points over all 10 patients is 2.02 ± 0.92 mm (Table 4).

**TABLE 4:** 3D vector difference between predicted and detected diaphragm tracking structure position during the tracking verification state.

Patient ID	Mean absolute deviation (mm)	(SD) (mm)
1	1.76	0.70
2	1.48	0.58
3	1.49	0.81
4	2.69	1.35
5	2.36	1.07
6	2.93	1.33
7	1.43	0.34
8	2.20	0.76
9	1.14	0.63
10	2.70	1.10
Mean	2.02	–
RMS of the SDs		
Random error	–	0.92
SD of the mean systematic error	0.64	–

#### 3.2.2 Film dosimetry verification of MDTT with diaphragm as surrogate

**FIGURE 10:**
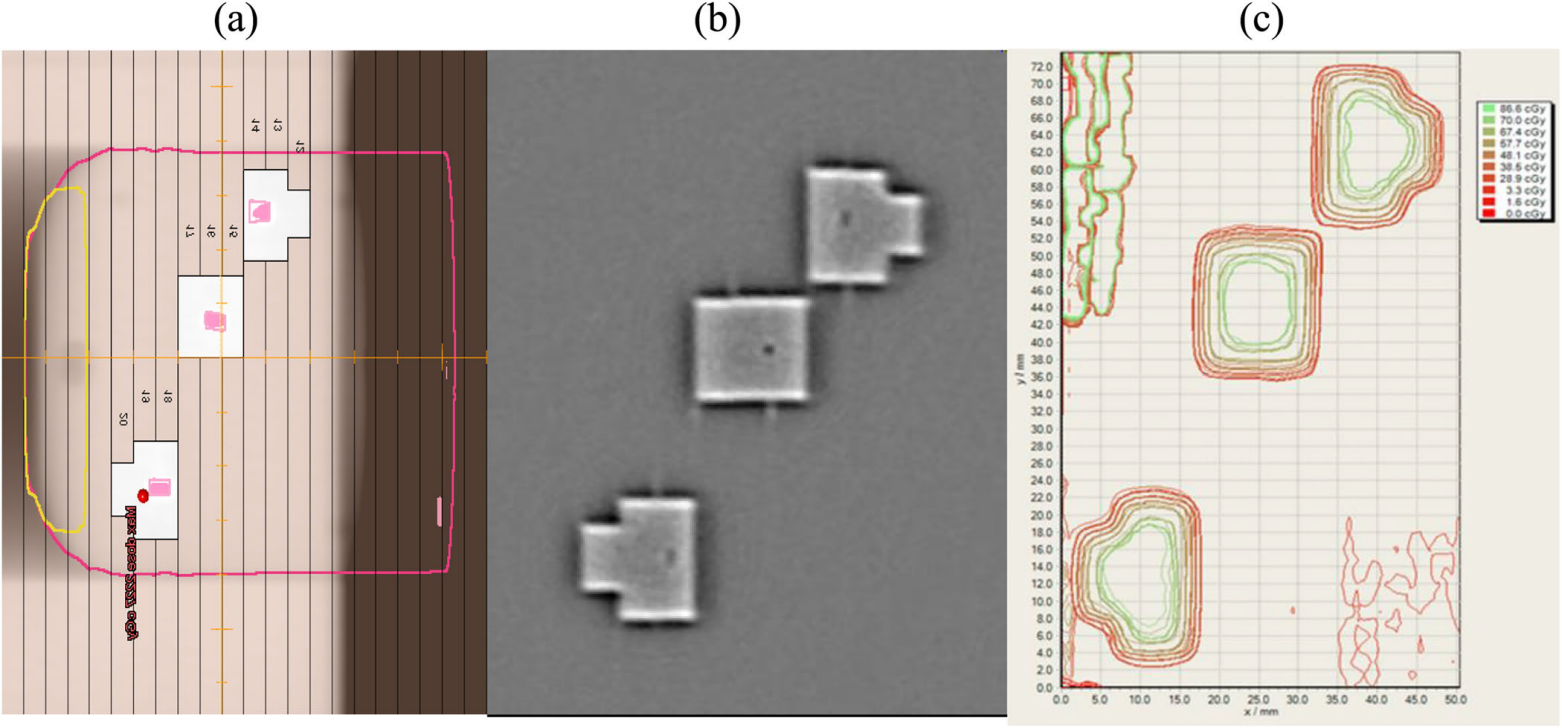
(a) The BEV view from TPS of the single field, multiple apertures plan. Note the fiducial within each aperture. (b) EPID image acquired during MDTT phantom delivery. (c) The film‐measured 2D isodose distribution comparing static‐field delivery (thin line) to MDTT delivery (thick line); lines overlap directly.

In phantom, a single field, multiple‐aperture plan was delivered under static and MDTT conditions. The treatment plan aperture and the resulting film analysis comparing the static‐field delivery to MDTT delivery is shown in Figure 10. The 2D gamma analysis for the film comparison (2%, 2 mm, 30% threshold) was calculated to have a passing rate of 98.2%.

#### 3.2.3 Planning target margin estimation

From Table 2, the accuracy of using the diaphragm as a surrogate for the fiducial markers is quantified. The SD of the systematic error for all liver patients was 0.34, 0.86, and 0.77 mm in ML, SI, and AP directions, respectively. The SD of the random error for all patients was 0.89, 1.26, and 1.06 mm in ML, SI, and AP directions, respectively. From Table 4, differences between the detected and predicted diaphragm COM position during dynamic tracking is reported. The 3D SD of the systematic error and the 3D SD of the random error for all patients during this tracking state was 0.64 and 0.92 mm, respectively. Using the methodology from van Herk,^48^ the contribution to the planning target margin due to these two sources of MDTT uncertainty is calculated to be 2.2, 5.0, and 4.7 mm in the ML, SI, and AP directions, respectively.

## 4 DISCUSSION

In this IRB‐approved imaging only patient study, a kV fluoroscopy‐based technique is used to assess liver tumor fiducial marker motion based on monitoring the diaphragm as a soft tissue surrogate for superior liver (or inferior lung) targets. The maximum distance between the diaphragm and the COM of the markers, measured in the superior–inferior direction, was 82 mm. The results of this study show that the mean ± RMS of the absolute difference between the COM of the markers position and COM of the diaphragm position was 0.70 ± 0.89, 1.08 ± 1.26, and 0.96 ± 1.06 mm in ML, SI, and AP directions, respectively. No correlation of the motion differences versus distance from the diaphragm was found. In comparison, Yang et al.^3^ used single‐slice cine‐magnetic resonance imaging simulation to assess the agreement between visible liver tumor and diaphragm motion in 2D views with a normalized cross‐correlation‐based template tracking technique. The mean difference values were 2.2 ± 0.5, 2.8 ± 1.4, and 2.4 ± 1.1 mm in the ML, SI, AP directions, respectively, for tumors up to 80 mm inferior to the diaphragm. This group did report a correlation between motion differences and distance from the diaphragm with a maximum difference of 4.6 mm in the superior/inferior direction at a location 8 cm from the diaphragm. Seppenwoolde et al.^20^ compared liver SBRT positioning methods, including markers and other surrogates (vertebrae, diaphragm, and dome of liver), using daily exhale breath‐hold contrast‐enhanced CT scans. The random/systematic liver tumor location prediction errors when using diaphragm as surrogate were calculated to be 1.6/1.2, 1.6/2.3, 1.5/2.1 mm in the ML, SI, and AP directions, respectively. In comparison, this study reports the diaphragm location verses markers random/systematic errors as 0.9/0.3, 1.3/0.9, and 1.1/0.8 mm in the ML, SI, and AP directions, respectively.

The Vero4DRT Markerless Dynamic Tumor Tracking module has been employed to assess the feasibility of tracking the diaphragm. Patient kV images demonstrate that the diaphragm provides enough contrast in kV fluoroscopy images for the Vero4DRT/ExacTrac system to detect and follow this tracking structure during respiration. A respiratory correlation model can be established, and the system was able to track this surrogate during the imaging‐only portion of this study. During gimbal‐motion dynamic tracking, the mean absolute difference ± RMS between the predicted and detected diaphragm location is 2.02 ± 0.92 mm.

Other groups have reported using kV images to track liver tumor positions using the diaphragm as a surrogate. Hirai et al.^49^ developed a new method to track tumor position using fluoroscopic images and a priori knowledge of the positional relationship between the diaphragm and the tumor calculated from four‐dimensional computed tomography (4DCT) data. They evaluated four metrics: diaphragm edge detection error, modeling error, patient setup error, and tumor tracking error, using data from seven liver cases. Overall, the mean positional error in tumor tracking in the fluoroscopy sequences was 1.30 ± 0.54 mm.

In a phantom study, Dick et al.^34^ used 4DCT datasets and the CyberKnife kV imaging/optical monitoring system to find the correlation between the location of the diaphragm and the 3D position of a liver tumor using artificial neural networks. This study concluded that the diaphragm could replace markers in the tracking of liver or other abdominal tumors. Hindley et al.^35^ proposed a method to achieve real‐time diaphragm monitoring on kV images acquired using a standard medical linear accelerator with the hope it would have applications for future real‐time, MLC‐based, tumor tracking applications. CT simulation‐generated 3D models of the diaphragm were projected onto 2D planes at various rotating gantry positions of the imaging x‐ray source (0.5° increments) to guide the structure localization on live kV images. Absolute errors (95th percentile) in the ML, SI, and AP direction ranged from 0.5 to 3.1, 1.6−6.7, and 1.2−4.0 mm, respectively, for six patients. Li et al.^32^ assessed the feasibility of using a diaphragm contour to guide and build a dynamic tracking correlation model for the Accuray CyberKnife system. In this patient tracking simulation study, the maximum distance between the diaphragm and the tumor location was 62 mm. They report an “average standard error”/“maximum standard error” of 1.8 ± 1.2 mm/1.9 ± 1.2 mm when the diaphragm was used as a surrogate.

The results of this current study can inform the PTV margin used for clinical implementation of this technique. Using the van Herk formulism, the contribution to this margin due to MDTT uncertainty is 2.2, 5.0, and 4.7 mm in ML, SI, and AP direction. As the markers are also a surrogate for the liver tumor, it is expected that the diaphragm‐to‐tumor uncertainty could be a higher than what is reported here. Seppenwoolde et al.^20^ suggest that the markers as a surrogate for the liver tumor could have an additional 3D random/systematic uncertainty contribution of 1.3/1.6 mm. However, compared to traditional motion encompassing methods, these margins will still result in a substantial decrease in PTV volume for the same GTV size. Dunne et al. reported a PTV volume reduction of 38.4 % (range 2.4–64.1) for DTT planning relative to the ITV motion encompassing method for 22 patients treated with liver SABR.^50^ The PTV margin used for the tracking patients in this study was 5 mm.

Although kV x‐ray imaging dose was not measured in this study, a comparison of marker‐based versus markerless dynamic tumor tracking can be estimated by referring to the literature. Ziegler et al. measured a CTDI of 0.033 cGy/image pair on the Vero4DRT (100k, 250 mA, 10 ms).^28^ Typical liver SABR DTT prescription doses at this center range from 900−1800 cGy/fraction (for 3 – 5 fractions), delivered with seven static‐field IMRT fields. On average, the correlation model building step acquires ∼35 image pairs and the treatment verification step acquires ∼85 image pairs per beam (at 1 Hz). This results in a CTDI dose for marker‐based DTT of approximately 20.8 cGy. Markerless DTT introduces an additional detection template mini‐fluoroscopy step (∼13 images) at each treatment beam which results in an additional 3 cGy of imaging dose. In the Ziegler et al. study, they compare their DTT imaging dose calculations to the CTDI value of CT guided marker implantation (∼14 cGy). The markerless DTT method could potentially remove the need for implanted markers for eligible patients, thus realizing a dose reduction of 14 cGy to the patient.

With respect to treatment times experienced by the patient, the MDTT workflow introduces two additional steps compared to traditional marker based DTT. First, the user must manually align the soft tissue tracking structure with one image pair from the correlation model fluoroscopy sequence (approximately 2 min). Second, for each treatment beam, a detection template is acquired and assessed (approximately 1 min per beam). For a seven‐field treatment plan, MDTT would require an additional 9 min of additional treatment time. At our center, a typical marker based DTT liver SABR delivery takes approximately 25 min. The MDTT workflow could increased that treatment time to 35 min, assuming the correlation model holds, and no model updates/rebuilds are required.

There are several limitations to this study. First, the patient imaging‐only study has ten patient datasets with a limited range of marker‐to‐diaphragm distances represented. It is difficult to ascertain if there is a correlation between marker (tumor) position uncertainties versus the marker distance from the diaphragm. Second, although many sources of uncertainty are quantified in this study, the list is not exhaustive. There is uncertainty in the detection of the true diaphragm contour, whether it be from the in‐house MATLAB analysis program, or from the clinical ExacTrac MDTT software. The detected diaphragm uncertainty relative to a gold standard, such as having a radiology expert define the interface on all kV images, was not investigated in this study. Other authors^49^ have quantified their kV image detection uncertainty against ``manually input reference diaphragm edge positions.’’ The diaphragm detection uncertainty in their study was estimated to be 0.57 ± 0.62 mm.

This study compares implanted marker positions relative to a proposed soft tissue surrogate for the liver tumor, namely, the diaphragm. Ultimately, the markers themselves are a surrogate for the liver tumor. Assessing the true location of the liver tumor relative to the diaphragm was not within the scope of this study.

Future directions include applying MDTT to other delivery methods, for example, dynamic conformal arcs, which are compatible with the MDTT module on the Vero4DRT. The clinical implementation process of this markerless diaphragm tracking technique for liver SABR DTT is currently underway.

## 5 CONCLUSION

The diaphragm motion correlated well with implanted COM‐marker positions for superior liver lesions up to 82 mm from the diaphragm and could be used as a reliable surrogate for motion. MDTT workflows using diaphragm as an MTS was feasible using the Vero4DRT linac. This MDTT method could replace invasive implanted marker‐based tumor tracking for eligible liver cancer radiotherapy patients.

## AUTHOR CONTRIBUTIONS

Study conception and design: Alanah Bergman, Roy Ma. Data collations: all authors. MATLAB code preparation: Maryam Rostamzadeh, Steven Thomas. Liver Phantom: BG, Maryam Rostamzadeh, Alanah Bergman. Data Analysis: Maryam Rostamzadeh, Alanah Bergman. Manuscript Preparation: Maryam Rostamzadeh, Alanah Bergman. Manuscript Revision/Editing: all authors.

## CONFLICT OF INTEREST

The authors have no relevant conflicts of interest to disclose.
